# It is not a big deal: a qualitative study of clinical biobank donation experience and motives

**DOI:** 10.1186/s12910-022-00743-6

**Published:** 2022-01-29

**Authors:** Natalia Antonova, Ksenia Eritsyan

**Affiliations:** 1 The Institute of Psychology, Herzen State Pedagogical University of Russia, 48, Moika Emb., Saint Petersburg, Russia 191186; 2 HSE University, 55/2 Sedova street, St. Petersburg, Russia 198099

**Keywords:** Biobank, Informed consent process, Motivation, Research ethics, Donation

## Abstract

**Background:**

The success of biobanking is directly linked to the willingness of people to donate their biological materials for research and storage. Ethical issues related to patient consent are an essential component of the current biobanking agenda. The majority of data available are focused on population-based biobanks in USA, Canada and Western Europe. The donation decision process and its ethical applications in clinical populations and populations in countries with other cultural contexts are very limited. This study aimed to evaluate the decision-making experience of the clinical biobank donors, as well as psychological and social motivators and deterrents of this decision and associated ethical risks.

**Methods:**

Semi-structured interviews were conducted in two medical institutions, in St Petersburg (Russia), in 2016–2017, among 13 donors of a clinical biobank (pregnant women, cardiac patients, and patients with multiple sclerosis) and three donation organisers—medical specialists involved in recruiting donors for a clinical biobank. Analysis of interview data was based on qualitative content analysis.

**Results:**

Donors of a clinical biobank express beliefs in the absence of risks associated with the donation. The primary motivators for donating to the biobank were: prosocial, indirect reciprocity (response to or anticipation of an act in kind by a third party), intrinsic motivation (to enhance their self-esteem and satisfying their curiosity about the donation process), and comparability with personal values. A high level of trust in biomedical research and the particular physician can contribute to a favourable decision. The overall decision-making process regarding the biobank donation could be described as quick and not based on a careful reading of informed consent documents. The integration of biobank donation decision-making in the process of medical care might prompt patient to donate to biobank without proper consideration. The specific type of therapeutic misconception—the presence of unrealistic hope that donation could provide a direct benefit for a third person in need was discovered.

**Conclusions:**

Patients recruited to a clinical biobank in Russia have virtually no concerns as to the storage of their biomaterials. The donation decision is mainly motivated by prosocial attitudes and other factors that are similar to the motivating factors of blood donation. The fact of going through inpatient treatment and poor differentiation between donation for other people's benefit and for research purposes can make the process of obtaining consent more ethically problematic.

**Supplementary Information:**

The online version contains supplementary material available at 10.1186/s12910-022-00743-6.

## Background

Today considerable efforts are being made to create biobanks—specialised storages of human biological samples and associated data for use in research and medicine. Biobanks are an important resource in public healthcare, as they are relevant for medical and biological studies, for meeting challenges of evidence-based medicine and developing new diagnostics methods and treatments, and other purposes [1, 2]. There are various classifications of biobank, as the term is quite new, and there is no single, universally accepted definition [3, 4]. According to the widely accepted classification, developed by Biobanking and Biomolecular Resources Research Infrastructure, biobanks are divided into two main types: (1) population biobanks (prospective biobanks with a focus on studying various populations or social groups); (2) clinical biobanks (banks of tissue samples and clinical data for the study of diseases) [5]. All residents of an area can be donors for the former group of biobanks, while those who suffer from specific conditions or who have clinical specificities can be donors for the latter group.

The term "biobanking" covers not only the biobank infrastructure itself, sample collection, biosample data, engineering, and technological solutions, but also extends to the whole range of social, legal, and ethical problems that must be resolved as biobanks develop [6–10]. Ethical concerns may incorporate compliance with recognisable standards, robust systems for stakeholder engagement, meaningful consent processes, strategic and authentic communication, and attention to the just distribution of research benefits [11]. Overall, the success of biobanking, whether population or clinical, is directly linked to the willingness of people to donate their biological materials for research and storage. Thus, the availability of voluntary donors is the cornerstone of biobanking [12]. In this regard, informed consent is one of the most discussed topics in the context of biobank research [13].

Most biobanks are located in the United States or Europe [14], which means that much of the information we know about the ethical, social, and psychological aspects of biobank recruitment may not be entirely applicable to biobank systems in other regions of the world.

What do we know about the biobank donors' motivation and informed consent process? There are some common biobank donors’ motives identified in the research literature—namely, altruism, reciprocity, an expectation of personal benefit through new therapies, direct feedback of study results, the clinical encounter surrounding the donation (e.g., a test or physical examination), and material compensation [15]. There is also evidence that donor decisions are strictly determined by the donor classification. Cohort studies show that representatives of the general population have a less favourable attitude to donations, compared with cancer patients, who registered the most interest, and relatives of deceased patients, who were least interested in donation. The more acute a person’s need for the results of medical research, the more likely they express positive attitudes towards donation [16].

Today, in Russia, biobanking is rapidly developing [17], while ethical and legal issues of biobanking, related to personal data protection and anonymization, informed consent, and biomaterial usage rights, have not yet received broader public discussion [18].

There is still minimal knowledge about Russian biobank donors' motivation and experience [18–20]. In 2014, a study among Russian University students showed a little awareness regarding biobanks (21%), together with a relatively high level of willingness of the Russian youth to donate to that (74%); a minimal impact of value orientations, and the lack of influence of socio-demographic variables on the willingness to donate [20]. The predictive factors of willingness to biobank donation, among Russian university students and researchers, were awareness of the biobank, approval of members of their close social environment, donation fees, and the type of biological material requested [21]. No studies on clinical biobank donations in Russia have been found.

The purpose of this paper is to study the process of giving informed consent to donate biomaterials to a Russian clinical biobank among patients of different nosological groups as well as to describe the motives for donation and associated ethical applications.

## Methods

Our study design was a qualitative study. A maximum variation sampling strategy was applied to recruit participants for the study [22]. We ensured that the sample of selected interviewees was heterogeneous [23]. Between May 2016 and June 2017, the semi-structured interviews (n = 16) were conducted in two medical institutions in St. Petersburg (Russia) among:

- 13 donors of a clinical biobank (D). Their average age was 41 years (ranging between 26 and 67 years) and 4 out of 13 were male. Donors belonged to the following nosological groups of patients: 1) gynaecological profile (pregnant women (n = 6): without abnormalities and with toxaemia;); 2) cardiological profile (myocardial infarction—acute coronary syndrome; n = 4), 3) patients with multiple sclerosis (n = 3). The education level of the respondents was different—just more than half (n = 9) reported having a university degree;

- 3 donation organisers (ORG)—medical specialists involved in clinical biobank donor recruitment (2 men and 1 woman). We included this group in the study since the donors’ sample might be skewed towards those who are more willing to collaborate or have more chances to be found in the medical institutions at the time of the data collection. Therefore, by providing information about their recruitment experience, the group of donation organisers here serves as a proxy for the (potential) biobank donors, including those who did not agree to donate and were unwilling or unable to participate in the interview.

Sampling of the donors and donation organisers was purposive. The inclusion criteria for donors for the study were: the fact of clinical biobank donation; that this donation was made no more than a week earlier; a donor belonging to one of the three nosological groups (women in labour, cardiological profile, patients with multiple sclerosis). For organisers, the inclusion criteria was being a medical specialist who supervised biobank recruitment in the particular nosological group.

Two female interviewers, with a degree in psychology, conducted semi-structured interviews. All interviews were tape recorded. Interviewers, themselves, made the transcripts of interviews. Two project staff members independently generated lists of secondary codes based on the transcripts. The lists were merged, following discussion, to define a single list of secondary codes. Interviewers and researchers were not affiliated to the medical organisations, where donors and organisers were interviewed. No relationship with participants was established before the study.

The interviews with donors were focused on was their experiences of biobank donation, interviews with organisers – on the practices and experiences of recruiting participants for the study. Interviewees provided their written consent and were assured that all provided information would be kept confidential. The interviews were semi-structured, with open questions being used, to encourage the interviewees to talk about their experiences in their own words. The interview guides are presented in Additional file 1.

The data was collected at the clinic. The conditions were created so that, besides the participants and researchers, no one was present at the interview. No interviews were repeated. During the research, audio recordings were made to collect data, and field notes were collected after the interviews. The average interview length was 15 min. Transcripts were not provided to participants for any further comment and/or correction.

The data was analysed, using qualitative content analysis, which consists of systematic search and description of the meanings of qualitative data of different types [24].

Since taxonomies for the biobank donation motivation are absent, we assumed that the closest possible analogue for which such taxonomies already designed could be a decision on blood donation. This type of prosocial behaviour is close in terms of being health-related and the type of procedures involved. At the same time, it considerably differs at least in several ways. First, in terms of when and who would receive the main benefits from the donation: the society, or a particular recipient (patient), second – in the amount of effort needed to provide the material (integrated or not in the course of treatment), third—in the incentives involved (usually no for biobank donors but might be something like lunch and days off for blood donors). Also, while blood donation has a long history and is well known by society members, people might have no beliefs and opinions about biobanks or be concerned about risks associated with biomaterials storage.

In this regard, we used the taxonomy of motives and deterrents to blood donation, by Bednall and Bove [25], as a starting point for building a code system. According to this taxonomy, donation motivators are prosocial motivation, perceived a need for donation, incentives, indirect reciprocity, intrinsic motivation, personal values, social norms and marketing communications. In addition to the pre-defined themes, emergent themes were explored and then added to the taxonomy.

## Results

We distinguished two main themes relating to the decision making experience of the clinical biobank donors. One theme pertained to the informed consent process and the circumstances of the donor decision-making, the other—to biobank donation motivators and deterrents. The donation organisers’ perspective is presented separately in the end of each theme.

I. Informed consent and the circumstances of the donor decision-making

*Informed consent process. *The information on the opportunity for biobank donation was provided to patients orally by the doctor in charge of their case, and, in other cases, by the medical staff of institutions, where potential donors were having inpatient treatment. In addition, the written informed consent form was provided.

All but one of the donors confirmed that they were offered an informed consent form. Depending on the disease, the informed consent form consisted of 3–7 pages. It consisted of standard sections including study purpose, participants, research methods, storage of biological material and process for transferring encoded materials, information about the absence of remuneration for participation and possible risks. In 7 cases, patients read the document on their own, five patients reported that it was read out orally by a medical specialist. It should be noted that, sometimes, patients claimed they examined the printed document cursorily and were inattentive. Various factors can affect the way a patient examines the document—these include personal factors (e.g., a developed approach to working with documents and contracts), and situational ones. Cursory reading was reported by those patients who had to sign many treatment-related documents during their treatment consultation (in- and out-patients)—and those documents were usually associated with more serious risks.

"I don't particularly read the documents recently, I read selectively what is written there as there was too much information to read," (D. 3).

"I read when I signed it, but I didn't pay attention to the information itself". "I had a lot of documents on treatment and something else," (D. 13).

In some cases, the informed consent on biobank donation was examined together with the discussion of other medical procedures.

"While discussing a surgical procedure, a caesarean section, we discussed other topics and spoke about documents" (D. 1).

Also, a donor's physical condition was an additional factor that affected how detailed they studied the informed consent form:

"while being in reanimation would you read the informed consent? - I had such a condition..." (D. 10).

Most of the informants remembered that the signing of informed consent itself preceded the donation. However, two donors (a woman in labour and a patient with multiple sclerosis) found it difficult to remember whether they had signed the informed consent form, and three donors (all belonging to the nosological group of women in labour) said they did not sign the informed consent form.

The informants noted that, when they got acquainted with the informed consent, they either understood everything, or they did not want to go into details. Several patients (9 out of 13) did not have questions after examining the informed consent form. Should patients have questions, they were directed to the medical staff of medical institutions; the answers of the specialists satisfied all the donors. The questions regarded either the type of the collected material, the possibility of being a donor with a particular disease in the past (hepatitis in childhood), and clarifications of who would be a donor—a mother or child (in the nosological group of pregnant women). The questions which donors asked were also related to the research and the project under which it was conducted.

The most common emotional reactions of donors, to the proposal to donate the biomaterial, were calm and indifference—eight respondents indicated thus.

"I treat it consciously, calmly, without any emotions," (D. 13).

Two donors noted that they felt interested.

Fears or hesitations were uncommon for patients, at the moment of deciding to become a donor. Only three donors mentioned them. Hesitations were associated with the childhood illness (hepatitis), which, according to the patient, could prevent her from donation, with fear of making a mistake that a newborn child should become a donor, fear of finding out bad news upon the medical examination results.

The time taken to decide to donate, for most donors, ranged from a few seconds to 15 min. Only one patient decided on donation, taking a full day. One of the patients (a woman with multiple sclerosis) associated the long time required by her, to decide on donation, with her hesitations and fears of the upcoming testing and its results.

"they simply said, I would undergo a medical examination, and I thought I hardly needed it. Do I really need to find out bad news? - I'd rather not know it" (D. 12).

In one case, a respondent said that she decided, in advance, to become a stem cell donor, and the proposal to become a biobank donor seemed, to her, a suitable replacement.

"When I give birth to a child, I need to call somewhere, I want them to take my placenta ... because when I came here in (name of the Institute) for an open-door day, there were representatives of (name of the Cell Bank). ... at that moment I decided I would be a donor", (D. 1).

While deciding on biobank donation, five donors sought advice. All respondents who sought advice were women (four women in labour and one woman diagnosed with multiple sclerosis) and they consulted their husbands.

"At that moment my husband was with me, he also agreed, I could say we made the decision together," (D. 2).

We can assume that, since any medical manipulation can be associated with a potential risk for the child, most pregnant women considered it important to take a shared decision with the future father and, perhaps, to share responsibility. A patient with multiple sclerosis consulted her husband, who is a medical practitioner, trusting his professional opinion. In cases where family members were nearby, taking advice did not require additional efforts from donors.

"Often people come to us with family members, we discuss this in the presence of family members. Sometimes family members make a decision for the patient, they say "you need it, participate" (ORG 2).

*Process and circumstances of donation.* Biobank donors of the gynaecological profile did not always clearly differentiate between the types of material taken. While donors diagnosed with myocardial infarction and multiple sclerosis clearly spoke about blood donation, almost all donors of the gynaecological profile reported donation of the umbilical cord and/or placenta, while one donor reported donation of the afterbirth (D. 9).

"Honestly, I don't remember. I just said, take it if you need it. A peace of placenta, venous blood, I think they took something from the cord ... Is it bad that I don't remember?" (D. 7).

"I remember they took a piece of placenta and a piece of cord... I'm not sure about the blood," (D. 1).

According to donation organisers, they collected maternal venous blood, umbilical cord venous blood and placental tissue from women with complicated pregnancies.

One cardiological profile donor in this study had negative service experience. This donor expressed his dissatisfaction regarding the process of blood drawing, referring to the nurses' incompetence and lack of expertise.

"Nurses there can't take your blood right, I had bruises everywhere, you know, both here and there. It was the only thing I didn't like. Both my arms were ruined. I also had to get shots later, but there was simply no place left," (D. 10).

Other donors did not express any dissatisfaction regarding the process of donation.

"No incidents, nothing, everything was on point," (D. 5).

*Awareness of the biobank purpose.* Informed consent form included quite generic description of the biobank, highlighting that it combines analysis of different biological materials to study causes and course of the disease including genetic contribution, as well as to develop new methods of diagnosis, prevention and treatment. Although eight donors pointed out that the purpose of a biobank is to store biological material for scientific research, donors often do not understand the purpose of this research.

"I didn't think about it, I didn't ask. I think they are going to do some research... I still don't understand why they had to do it. I don't know what they are going to do next, or what for," (D. 3).

"Someone needs it for their thesis," (D. 9).

Clinical biobank donors do not always differentiate the research goal from the goal of providing direct help to a specific person in need of donor blood or an organ.

"When something happens to a person and they need to find a donor quickly, first of all they turn to this biobank," (D. 1).

"Q: So you think this biological material can be useful to somebody?

A: Yes, I hope it can. I mean, I hope nobody will need it, but if one day someone does, it will be great to know that it was useful and it happened to be at the right time at the right place," (D. 1).

"I guess it's that place where they store all this for several years and then use it for other people ... they use cord blood for treatment of some forms of cancer, there were many successful surgeries," (D. 6).

Considering the positive connotation of the term, donation, (as in blood donation) in Russian culture, one patient called donation to a biobank "passive donation", since it does not result in saving lives directly.

"We are not talking about saving lives here ... it's some kind of passive donation," (D. 7).

*Awareness of the potential risks.* The informed consent form includes standard information about health-related risks related to medical procedures such as blood sampling. A special section also was devoted to the issue of data storage and protection.

Nevertheless all biobank donors clearly stated their opinion that biobank donation is not associated with any risks and revealed no concerns regarding data storage and protection.

"I'm not concerned about the process, in fact. It doesn't hurt, you don't lose anything," (D. 8).

Only one respondent expressed certain concerns about the risks associated with the process of taking the biological material.

"What if I get infected? I also thought about it, actually," (D. 3).

*Organisers’ perspective*. Data received from organisers generally mirrors the one provided by donors. According to their experience, the typical recruitment takes 5–10 min, including giving information by the recruiter, patient’s familiarisation with informed consent forms, and questions and answers. Patients might not read forms carefully but mainly browse through them. Questions, if any, were mainly related to the procedural aspects of the study (timeframe and concrete tests).

II. Biobank donation motivators

The majority of the patients had not previously considered becoming donors; they made a spontaneous decision and, therefore, found it difficult to identify any predominant motivation.

"Q: What was the main reason you agreed to donate your biological material for research purposes? A: No reason, I just agreed, that's it... It was purely spontaneous," (D. 10).

"There was no special reason. They offered to do it and I agreed," (D. 11).

*Intrinsic motivation. *The donors who mentioned the presence of intrinsic motivation most often cited certain forms of prosocial motivations; moreover, the respondents frequently mentioned several co-existing motives without distinguishing between them. Altruism (desire to help other people) and collectivism (desire to help the community) were the most frequently reported motivations for donation:

"If I don't need it, maybe someone else will ... I'll do a good thing for somebody," (D. 1).

"Why not ... if it is beneficial to society," (D. 1).

"Besides, it may help people find out how to fight this disease," (D. 3).

In some cases, respondents indicated that either science or the healthcare system are the main beneficiary of their donation:

"It will help to develop our healthcare ... I don't mind if it's useful for some research," (D. 2).

"It’s useful, it helps to develop science ... why not contribute to the development ...“ (D. 9).

One donor also indicated that she had donated because she perceived a need for such service:

“You always see messages like ‘Help save this child,’ ‘Save this child!’ ... When you see mothers who face such a problem, you start praying that the same doesn’t happen to your family. And if you can do something to prevent it, then why not,” (D. 1).

Indirect reciprocity, defined as a “response to or anticipation of an act in kind by a third party”, was also a commonly cited motivator of donation behaviour. One donor indicated that she had donated out of gratitude (upstream reciprocity) after her family member and she, herself, had received medical treatment or lost their loved ones:

“If I can help someone, I will do it with great pleasure, because I know how hard it is to lose your loved ones ... My son also has a severe heart defect, he had undergone surgery,” (D. 13).

Some donors indicated that their donation behaviour was encouraged by other sources of intrinsic motivation. In particular, they cited feeling good about themselves (i.e., to enhance their self-esteem) after donation:

“I even thought, later, what a good thing I’d done,” (D. 7).

“There was awareness that I contributed to the development of science,” (D. 2).

And they were satisfying their curiosity about the donation process (i.e., an impulse to investigate, observe, or gather information, particularly when the experience is novel or interesting):

“I’m always curious to try something new,” (D. 1).

“I am always ready for new experiences,” (D. 3).

Two donors indicated personal values as their motivation to donate:

“This is such a noble goal that few people would give up,” (D. 2).

“I had already made a decision that I would become a donor in any case,” (D. 1).

Four donors reported donating because of perceived social norms. Even though donation to a biobank is a relatively new social phenomenon, respondents could relate it to a broader range of actions classified as prosocial behaviour. Thus, several donors were motivated to donate because blood donation was widespread among their friends and/or family:

“My friends and relatives ... they donate blood regularly ... I can’t be a blood donor but at least my pregnancy could help someone,” (D. 1).

“My father has received an award for being a blood donor for many years, and my cousin too,” (D. 3).

Donors indicated that they were encouraged to donate by other significant people or felt social pressure to donate (subjective norms).

“I was talking to my sister ... she said it (blood) replaces itself,” (D. 3).

“Everyone tells me, ‘You did the right thing!’ ” (D. 5).

“My husband is a physicist, he also studies medicine and he understands all this ... My husband is a scientist, he has been doing research all his life, he probably needs it, so I believe him,” (D. 12).

The study results allowed us to identify two categories of motives that were not included in the list proposed by Bednall and Bove [25]. They are unwanted / low-value gifts and the level of trust in healthcare and a particular physician.

Five donors summarised their motive for making a donation in a sentence “I don’t need it.” In cases where pregnant women donated their placental tissue, they could perceive it as “waste material” of no particular significance [15] which is removed during medical interventions and usually disposed of.

Thus, donation to a biobank may be perceived as obtaining additional benefits for someone without any additional costs to oneself.

“You don’t need it anyway. You will throw it away otherwise,” (D. 1).

“I just don’t need it ... if someone needs this material, let them take it,” (D. 6).

In some cases, a high level of trust in biomedical research, healthcare, and particular physicians can encourage people to decide to become biobank donors. Patients delegate to their doctor the duty to analyse data about the importance and safety of the biobank donation and make a positive decision based on this analysis and the level of trust in their doctor.

“I understood that they were going to use it for some good purpose, it was enough for me because I trust my doctor,” (D. 1).

“I believe they won’t deceive me, they won’t remove my organs or anything,” (D. 3).

“I decided to give them a chance, I just thought I could trust them,” (D. 7).

Several respondents (n = 6) answered the question about motivation, saying the same phrase: “if it has to be done, it has to be done” or “it’s not a big deal” (like here “it’s not a big deal to me, if they need this material, why not?” (D. 6)). In Russian, it is a set expressions that can be explained as a willingness to comply with some external requirements or requests without delving deeper into the matter.

*Incentives*. Biobank donors mostly stated that they had received no rewards or direct benefits in the form of financial rewards, time off work or gift items for making donations:

“They told me at the very beginning, there are no benefits... the doctor warned me there would be no compensation, no incentives at all,” (D. 5).

“I think no, I understood it was a voluntary donation without any incentives,” (D. 6).

However, some of the biobank donors either found it difficult to answer the question about incentives or pointed out some benefits for themselves, mainly in the form of health checks that are free and not time-consuming.

“A full check-up ... I wouldn’t have done a check-up myself otherwise,” (D. 4).

“... to do a full check-up. It’s rather expensive nowadays ... And they told me it was free there ... to examine my health ... just for myself ... It would be stupid to refuse,” (D. 5).

One donor indicated perceived health benefits—a belief that blood donation will provide positive health effects.

“When you donate blood, it replaces itself later. It’s good for your health,” (D. 3).

Several donors who answered the question “Do you want to know how your samples and information are being used?” expressed their interest in obtaining the results of the research.

“I think everyone is interested. I’d like to know,” (D. 3).

Nevertheless, patients demonstrated rather moderate interest in receiving feedback on the results of their participation in the biobank.

“I don’t want to impose myself... What’s there?” (D. 2).

“It would be interesting but it’s not my area of expertise, so I probably won’t understand anything,” (D. 9).

“I think they include this information into your medical history and your physician reads it later,” (D. 5).

“As for now, it is still being developed, I think. I don’t know what they are going to do next,” (D. 6).

“It was more important to me to have a healthy baby,” (D. 7).

It should be noted that some donors emphasised that they were interested in obtaining only their results, rather than results concerning other people.

“My results, not someone else’s,” (D. 12).

“I’d like to know about my blood values,” (D. 11).

It was the lack of personalised data that patients used as the main reason for not being interested in obtaining research results.

“What kind of results? Why should I be interested in this? Why are heart attacks more common in people with brown eyes than in people with blue eyes?” (D. 10).

One respondent indicated that he had become interested in research results long after making the decision on becoming a donor and the donation itself.

“But now I’ve started asking questions like, ‘What is the purpose of all this? What is going to come out of it?’ ” (D. 3).

*Organisers’ perspective. *Again, data received from organisers mostly agree with one provided by donors. Despite the fact that patients usually heard about the biobanks for the first time while being asked to donate, the overall majority agreed. And it seemed natural and ordinar for organisers. Two predominant motives for the agreement were mentioned. The first one is the incentive motivation in the form of additional, otherwise costly check-ups and organisers specifically emphasised it.

Q: Where there any reactions - surprise, irritation….?

R: no, the surprise is that all this is free for them

Q: ah ... they are surprised that it is free?

R: yes, "I really have to pay?" - "Really?", "really?" - "really!",” (ORG 2).

“…they receive a complete medical examination that they would not have received in life,” (ORG 3).

The other motivation wasn’t pronounced by donors, but was perceived as an important one by some organisers is the moral obligation (as a kind of personal value according to Bednall and Bove classification [25]), to participate in research for patients receiving medical aid in organisations which have a status of “research institute”.

“They realize they are in a research institute. … yes, they are immediately calm, they understand what is needed for science” (ORG 1).

## Discussion

The study results indicated that unlike patients in many Western countries [26–29] donors of a clinical biobank in Russia do not express negative attitudes towards biobanking and do not voice any fears or concerns related to the genetic research. The donation organisers also confirm the absence of any comprehensive difficulties related to the recruitment of donors. We believe this might be due to lack of public discussion of biobank idea in Russia – the recruitment process might be first time when participants heard about biobanks.

Some authors believe that informed consent does not serve to protect the interests of donors to the full extent, since donors rarely, if ever, read it and use the information obtained from it [16]. A systematic review of the studies, discussing the informed consent processes in human genetic studies [30], revealed that about 93% of participants spend less time reading their informed consent form than the minimum estimated reading time. They did not read its sections carefully and, consequently, did not always comprehend its contents. E. Jacquier et al. found that most of the informed consent forms were too complex to be fully understood by most of the potential research participants [31]. In the systematic review, focused on the biobank participants, D’Abramo et al. [13] have also found lack of recall and understanding of the informed consent form. The findings of the current study have shown similar results. The main reasons for reading the informed consent form cursorily were directly related to the clinical type of biobank donation. First, donors do it along with signing multiple medical documents that are related to treatment and associated with more serious risks; second, their physical condition and state of mind are affected by an illness and the treatment process.

European studies show that participants are motivated to do good deeds by helping researchers, patients, and doctors [32]. According to the review data, obtained by D. Budimir and her colleagues [33], many potential donors often fail to comprehend the purpose of a biobank and its research objectives, but are eager to become donors. This phenomenon has been confirmed in our study as well.

Respondents generally make a donation decision independently, although the opinion of significant others stimulates them when they make such a decision. In the case of pregnant women, a partner’s approval is an important factor encouraging the donation decision, which can be explained by the desire to share responsibility for the possible risks to the health of the child with the father.

Overall as we expected motivation for biobank donation to large extend comparable to those of blood donation [25]. The main motivators for donating to the biobank were prosocial motivation, indirect reciprocity, intrinsic motivation (to enhance their self-esteem or satisfy their curiosity about the donation process), and personal values. Donors’ motivation to receive additional check-up options as a form of incentive motivation was perceived as predominant by donation organisers. This finding might be ethically challenging given a high chance for receiving incidental findings, especially with particular methods and populations [34]. In our study, "full check-up" was not perceived as a potential risk by organisers or donors, which might be attributed to the novelty of such practice in Russia.

However, several additional types of motives have been identified. First, donating “waste” biological materials (e.g. placental tissue) can be regarded by some donors as an unwanted/low-value gift [9]. Participation in the research in this case could be hampered only by procedural aspects (especially by time costs), which are neutralised by the conditions of a hospital stay. Second, a high level of trust in biomedical research, medicine and a certain physician can contribute to the positive decision to donate. In this case, patients use their trust to those institutions as a shortcut to the decision-making and transfer it to the biobank.

Our study has revealed that patients insufficiently differentiate between the actual clinical and research objectives of biobanking. Not all the donors clearly comprehended what are the outcomes of biobank, being more interested in obtaining the results of personal medical examination. A systematic analysis by Nobile et al. [35], indicated that the process of making a decision on biobank donation often linked to the expectation of personal benefit, through health-related information (or health check)—is recognised by some authors as a therapeutic misconception. As of now, the scientific literature has relatively little data on the phenomenon of therapeutic misconception (or its equivalent in the context of genetic studies—diagnostic misconception), insofar as making a biobank donation decision is concerned. However, the magnitude of this phenomenon is an important ethical concern, as misconceptions can lead to the formation of distorted motives to donate. Thus, it remains an open question, whether the expectation of a health check, when donating to a biobank, is a manifestation of therapeutic misconception or a type of incentive. It is especially interesting, in light of the fact that the donation organisers, themselves, are confident that biobank donation is a unique opportunity that patients should use.

Our study also identified a phenomenon that can be described as a “beneficiary for others misconception”—i.e., an unrealistic expectation that donation could give instant advantages to a particular third person. Such a misconception might be facilitated by the fact that the term “donation” itself, as applied to biobanking, draws upon the positive connotation of the term “donation” used in other contexts (e.g., blood donation), which is widespread in Russia. Therefore the prosocial motivation of some donors might be based on the wrong understanding of the biobank functioning. This appears to be a promising topic for further study.

We can, therefore, conclude that the process of deciding on donation to a clinical biobank can take a short time and be based on several factors: the attitude to donation in general; confidence in medicine, a particular hospital or physician; and risk assessment. Certain ethical risks arise from the fact of a donor having to make a decision while undergoing medical treatment, since a patient has to simultaneously make treatment-related decisions that are more personally important and have a higher priority. This distorts the process of making a decision to donate, or prompts making a biobank donation without proper consideration.

Several practical implications could be proposed based on this analysis. First, the content of informed consent in settings where the idea of biobank is relatively novel should be elaborated. Particularly it should ensure to provide correct ideas about the biobank, including how soon and which way society and other patients might benefit from its activity and for how long the specimen would be stored. Second, while patients worldwide might indeed consider additional check-up as an attractive incentive, it should be proposed together with the proper discussion of risks and management of incidental findings. Risks of potential receiving of incidental findings should be adequately communicated to potential donors alongside potential benefits [36]. Overall, since the decision of clinical biobank donor is facilitated through established rapport with the healthcare setting and is distorted via being done together with clinical decision, special efforts should be made to ensure the informed consent quality. Provision of more interactive forms of informed consent materials [37], providing time breaks between different medical-related decisions, and introduction of short quizzes to test participants' understanding might be potentially good solutions.

Our study has several limitations. First, it included only those patients who agreed to become biobank donors—so we see only the perspective of such patients, but not of those who refused. According to donation organisers, only a small number of potential donors refuse to donate biomaterials to a biobank. Unfortunately, no information about the reasons for such decisions has been collected nor by those who recruit, nor by institutions. However, the perspective and decision-making specifics of those who decline are of considerable interest. Second, this study was based on the data obtained from three groups of patients with different nosology. We were able to identify only some differences between these groups—in particular, the shared decision-making of pregnant women and their partners, and, also, the attitude of pregnant women to the biomaterial that is perceived by them as an unwanted gift, that will anyway be taken and otherwise would simply be disposed of. There is a reason to assume that the characteristics of the disease, the types of manipulations carried out and the social characteristics of patients can influence the process and the result of their donation decision—the research in this sphere might be extremely promising. However, to our best knowledge, it is the first study conducted in Russia focused on the process and factors of making a decision to donate to a biobank among various groups of patients.

## Conclusion

Patients recruited to a clinical biobank in Russia have virtually no concerns as to the storage of their biomaterials, unlike patients in many other Western countries. The decision on donation is mainly motivated by prosocial attitudes and other factors that are similar to the motivating factors of blood donation. Our study identified that there is a misconception, based on the unrealistic hope that donation could give instant advantages to a particular third person, in the same way as donating blood might help a person in need of blood transfusion. An additional factor contributing to donor consent, is the notion that materials coming to a biobank are an undesirable or low-value gift, and that they would otherwise be disposed of. The fact of going through inpatient treatment can make the process of obtaining the consent more ethically problematic, since, firstly, it is often accompanied by making decisions concerning other medical procedures which are directly related to a patient’s health and are more personally significant, and, secondly, the consent is facilitated by a patient’s trust in the hospital’s medical personnel that recruits donors to a biobank. Using additional check-ups as an incentive motivation might be suboptimal from the ethical perspective. Improving quality of decision-making and informed consent process among clinical biobank donors requires additional actions especially in settings where awareness about biobank is low.

## Supplementary Information

**Additional file 1:** The interview guides.

## Data Availability

The datasets generated and/or analysed during the current study are not publicly available due to confidentiality requirements, but are available from the corresponding author on reasonable request.
