# Supplementary material for: It is not a big deal: a qualitative study of clinical biobank donation experience and motives
Source: BMC Med Ethics. 2022 Jan 29;23:7. doi: 10.1186/s12910-022-00743-6 (PMC8800256; doi:10.1186/s12910-022-00743-6)
Supplement: Supplementary file 1 — Additional file 1: The interview guides. [file 12910_2022_743_MOESM1_ESM.docx]

The interview guide

# Date of interview_______________

Date of sampling and type of biomaterial ______________

Health Condition ____________

Sex (male/female) (underline)

1. Have you ever participated in clinical studies before? Have you ever been a donor? Tell us in detail, please.
2. Tell us in detail who provided you with the information on the opportunity to become a donor to a biobank and by what means (a physician (what is his/her specialization?), in writing, during a private conversation, in an advertisement, etc.)
3. Were you given the informed consent (IC) form? Did you read it yourself? Or did a medical specialist read it to you? Do you think you read the IC form carefully? Was everything clear to you in the IC form? Was the IC form written in plain and understandable language? Did everything seem correct to you? Did you have any questions after reading the IC form? If yes, what were they? Did you ask those questions to a researcher? What reply did you get? Were you satisfied with the answers?
4. Can you recall your reaction when you were suggested to donate biological material (for instance, you felt surprised, annoyed, angry, enthusiastic, etc.)? Why did you feel that? Did you hesitate or feel fear while deciding to become a donor? (A hint to the interviewer: specify the fears and hesitations)
5. How much time did it take to decide that you would become a donor to a biobank? Many people consult others while making such a decision. Did you consult anyone? (Do not pronounce: relatives, physician, the internet)?
6. If you were asked to tell a friend what a biobank is and what the purpose of the research in which you participated was, what exactly would you tell him/her? Use your own words. (A hint to the interviewer: if the response does not cover the goals of a biobank, the research for which biological material was sampled, type of biological material, benefits for donors, and risks that participants take, ask about it)
7. What was the main reason you agreed to donate your biological material for research purposes?
8. Are you interested in getting access to the results of the research? Did you receive a reward/compensation for donating to the biobank, whether material or not (a badge, a day-off, etc.)?
9. In your opinion, why do people refuse to donate to a biobank? Is there anything else you consider important to share with us on your donating experience that we did not ask you about?

And finally, a few questions about you.

- Age
- Citizenship
- Education
- Health condition

Thank you for cooperation!
